# Hepatic CEACAM1 Over-Expression Protects Against Diet-Induced Fibrosis and Inflammation in White Adipose Tissue

**DOI:** 10.3389/fendo.2015.00116

**Published:** 2015-08-03

**Authors:** Sumona G. Lester, Lucia Russo, Simona S. Ghanem, Saja S. Khuder, Anthony M. DeAngelis, Emily L. Esakov, Thomas A. Bowman, Garrett Heinrich, Qusai Y. Al-Share, Marcia F. McInerney, William M. Philbrick, Sonia M. Najjar

**Affiliations:** ^1^Center for Diabetes and Endocrine Research, College of Medicine and Life Sciences, University of Toledo, Toledo, OH, USA; ^2^Department of Physiology and Pharmacology, College of Medicine and Life Sciences, University of Toledo, Toledo, OH, USA; ^3^Department of Medicinal and Biological Chemistry, College of Pharmacy and Pharmaceutical Sciences, Toledo, OH, USA; ^4^Section of Endocrinology and Metabolism, Department of Internal Medicine, Yale University School of Medicine, New Haven, CT, USA

**Keywords:** insulin clearance, liver-adipose tissue axis, insulin resistance, steatosis, CEACAM1, fibrosis, inflammation

## Abstract

CEACAM1 promotes insulin extraction, an event that occurs mainly in liver. Phenocopying global *Ceacam1* null mice (*Cc1^–/–^*), C57/BL6J mice fed a high-fat (HF) diet exhibited reduced hepatic CEACAM1 levels and impaired insulin clearance, followed by hyperinsulinemia, insulin resistance, and visceral obesity. Conversely, forced liver-specific expression of CEACAM1 protected insulin sensitivity and energy expenditure, and limited gain in total fat mass by HF diet in L-CC1 mice. Because CEACAM1 protein is barely detectable in white adipose tissue (WAT), we herein investigated whether hepatic CEACAM1-dependent insulin clearance pathways regulate adipose tissue biology in response to dietary fat. While HF diet caused a similar body weight gain in L-CC1, this effect was delayed and less intense relative to wild-type (WT) mice. Histological examination revealed less expansion of adipocytes in L-CC1 than WT by HF intake. Immunofluorescence analysis demonstrated a more limited recruitment of crown-like structures, and qRT-PCR analysis showed no significant rise in TNFα mRNA levels in response to HF intake in L-CC1 than WT mice. Unlike WT, HF diet did not activate TGF-β in WAT of L-CC1 mice, as assessed by Western analysis of Smad2/3 phosphorylation. Consistently, HF diet caused relatively less collagen deposition in L-CC1 than WT mice, as shown by Trichrome staining. Coupled with reduced lipid redistribution from liver to visceral fat, lower inflammation and fibrosis could contribute to protected energy expenditure against HF diet in L-CC1 mice. The data underscore the important role of hepatic insulin clearance in the regulation of adipose tissue inflammation and fibrosis.

## Introduction

Carcinoembryonic antigen-related cell adhesion molecule 1 (CEACAM1) promotes insulin extraction in liver to regulate insulin action. This finding is bolstered by the observation that mice with liver-specific inactivation of CEACAM1 (L-SACC1) or with global null deletion of Ceacam1 gene (*Cc1^–/–^*) manifest hyperinsulinemia secondary to impaired insulin clearance. This causes insulin resistance in addition to hepatic steatosis (1–3). The latter is mediated by the transcriptional activation of lipogenic genes by sterol regulatory element-binding protein (SREBP-1c) in response to chronically elevated levels of insulin (4). It can also be caused by blunted CEACAM1-mediated downregulation of fatty acid synthase (Fasn) activity under hyperinsulinemic conditions (5).

Both L-SACC1 and *Cc1^−/−^* mutant mice also display visceral obesity (1–3). Contributing to this metabolic anomaly, is increased redistribution of hepatic triacylglycerol to white adipose tissue (WAT), in particular that mice are propagated on the C57/BL6J genetic background, which favors redistribution to adipose tissue (6).

We have recently shown that high-fat (HF) intake causes insulin resistance in part by reducing CEACAM1 mRNA and protein levels in liver (7). Conversely, transgenically protecting hepatic CEACAM1 in L-CC1 mice prevented diet-induced insulin resistance and limited hepatic steatosis in response to HF diet (7). Additionally, it blunted the negative effect of HF diet on energy expenditure and spontaneous locomotor activity. This protective effect of hepatic CEACAM1 gain-of-function could be mediated, at least in part, by inducing FGF21, which in turn, enhances fatty acid oxidation in liver (8, 9) and increases brown adipogenesis (10, 11).

L-CC1 transgenic mice with liver-specific gain-of-function also accumulated less total fat mass than their wild-type (WT) counterparts in response to 4 months of HF intake (7). Because CEACAM1 is barely detectable at the protein level in adipose tissue (12), we hypothesized that extra-adipocytic factors brought about by overexpressing CEACAM1 in liver are involved. To test this hypothesis, we investigated whether altered CEACAM1-dependent insulin clearance pathways regulate adipose tissue biology in response to HF diet.

## Materials and Methods

### Mice generation

As recently described (7), L-CC1 transgenic mice with liver-specific over-expression of FLAG-tagged WT rat CEACAM1 were generated using human apolipoprotein A1 (APOA1) promoter/enhancer element (13), and propagated on the C57/BL6J background (Jackson Laboratories), as described (7). The “minigene” construct was obtained by subcloning the proximal APOA1 promoter 5′ of a *Ceacam1* rat minigene-containing intron 1 into pCMV-3Tag-3A plasmid at *Not1* site.

Starting at 2 months of age, male mice were fed *ad libitum* either a standard diet (12:66:22% calories from fat:carbohydrate:protein) or a HF diet (45:35:20% calories from fat:carbohydrate:protein) (Catalog #D12451, Research Diets). Mice were kept in a 12-h-dark/light cycle. This study was carried out in accordance with the recommendations of the Institutional Animal Care and Utilization Committee (IACUC), which approved all procedures.

### Metabolic parameters

Mice were fasted overnight and their retro-orbital venous blood drawn at 11:00 hours the following day. Plasma was stored at −80°C until levels of insulin (Linco Research), FFA (NEFA C, Wako), and triacylglycerol (Pointe Scientific Triglyceride, Canton) were assessed. Hepatic triacylglycerol content was measured as previously described (1, 3).

### Body composition

Whole body composition was assessed by nuclear magnetic resonance (NMR; Bruker Optics).

### Immunofluorescence of visceral white adipose tissue

Visceral WAT was formalin-fixed, cut into 2–3 mm sections, and transferred to Dulbecco’s phosphate buffered saline (PBS, Sigma Aldrich) for 48 h at 4°C. Adipose tissue was permeabilized in 1% Triton X-100 (Fisher) in PBS for 10 min before being stained with rat anti-mouse F4/80 (Invitrogen, Carlsbad) to mark macrophages and detected with donkey anti-rat IgG conjugated to Alexa Fluor488 (Invitrogen). Tissues were incubated with primary antibodies overnight at room temperature (RT) in the dark and washed 3× with staining buffer before being subjected to secondary stain for 2 h and wash (3×) in PBS. Stained samples were then counterstained for 15 min at RT with 5 μM BODIPY 558/568 (Molecular Probes, Inc.) to visualize lipid. Adipose tissue samples were placed directly on a coverslip with buffer and visualized.

### Laser-scanning confocal microscopy

Samples were imaged using a Leica TCS SP5 laser-scanning microscope (Leica Microsystems) equipped with conventional solid state and a Ti-sapphire tunable multi-photon laser (Coherent). Images were acquired in the 3D XYZ plane in 2.5 μm steps with a 20× objective (NA 0.70) using the sequential scan mode to eliminate any spectral overlap in the individual fluorophores. Specifically, AlexaFluor488 (Invitrogen) was excited at 488 nm with collection at 500–558 nm. The BODIPY 558/568 dye (Molecular Probes, Inc.) was excited at 561 nm and collected at 567–609 nm. Selected images are a 2D representation of the 3D laser-scanning confocal microscopy (LSCM) image *z*-stack, as labeled.

### Gomori’s trichrome staining

Adipose tissue (*n* = 5 per mouse group) was fixed in 10% formalin and replaced by 70% ethanol before undergoing blocking in paraffin. Sections were deparaffinized at 60°C and hydrated in deionized water. Antigens were unmasked in Bouin’s Fluid at 56°C for 45 min. Upon rinsing in deionized water, nuclei were stained with Working Weigert’s Iron Hematoxylin at RT for 10 min. Trichrome stain was performed using the Thermo Scientific Richard-Allan Scientific Chromaview-advanced Testing (Cat. No. 87020). In brief, rinsed slides were trichrome stained at RT for 15 min and dehydrated sequentially in 1% acetic acid solution for 1 min, 95% ethanol for 30 s, and 100% ethanol for 1 min (twice). Sections were then cleared in three changes of clearing reagent for 1 min each and mounted.

### Western analysis

Tissues were lysed and proteins analyzed by SDS-PAGE followed by immunoprobing with polyclonal antibodies against Fasn (α-Fasn) (Assay Designs), fatty acid translocase/cluster of differentiation protein 36 (FAT/CD36) (Santa Cruz Biotechnology), apolipoprotein B (Chemicon International), and total and phospho-Smad 2 and Smad 3 (Cell Signaling). For normalization, monoclonal antibodies against α-actin (Santa Cruz) were used. Blots were incubated with horseradish peroxidase-conjugated anti-goat IgG (Santa Cruz Biotechnology), anti-mouse or anti-rabbit IgG (Amersham) antibodies, and proteins detected by enhanced chemiluminescence (ECL; Amersham) prior to quantification by densitometry (Image J software).

### Semi-quantitative real-time RT-PCR

Total hepatic RNA was isolated with PerfectPure RNA Tissue Kit (5′), and total adipose tissue RNA was isolated with RNeasy Lipid Tissue Mini Kit (Qiagen) according to the manufacturer’s protocol. cDNA was synthesized by ImProm-II™ Reverse Transcriptase (Promega), using 1 μg of total RNA and oligo dT primers (Table 1). cDNA was evaluated with real-time quantitative PCR (Step One Plus, Applied Biosystems). The relative amounts of mRNA were calculated by comparison to the corresponding standards and normalized relative to 18S.

**Table 1:** **Real-time PCR primer sequences from mouse genes**.

Primer	Forward sequence (5′–3′)	Reverse sequence (5′–3′)
Cd36	TCTTGGCTACAGCAAGGCCAGATA	AGCTATGCATGGAACATGACG
Fatp-1	TCACTGGCGCTGCTTTGGTT	GGACGTGGCTGTGTATGG
Fatp-4	GTGAGATGGCCTCAGCTATC	GAAGAGGGTCCAGATGCTCT
Lpl	AAGGTCAGAGCCAAGAGAAGCA	CCAGAAAAGTGAATCTTGACTTGGT
Srebp-1c	GGAGCCATGGATTGCACATT	GCTTCCAGAGAGGAGGCCA
Hsl	GGCTTACTGGGCACAGATACCT	CTGAAGGCTCTGAGTTGCTCAA
F4/80	CAAGGAGGACAGAGTTTATCGTG	CTTTGGCTATGGGCTTCCAGTC
TNFα	CCACCACGCTCTTCTGTCTAC	AGGGTCTGGGCCATAGAACT
Smad7	GTTGCTGTGAATCTTACGGG	ATCTGGACAGCCTGCA
Col6α3	ACCTAGAGAACGTTACCTCACT	GTCAGCTGAGTCTTGTGCTGT
α-Sma	CGTGGCTATTCCTTCGTTAC	TGCCAGCAGACTCCATCC
18S	TTCGAACGTCTGCCCTATCAA	ATGGTAGGCACGGCGACTA

### Statistical analysis

Data were analyzed with SPSS software by two-way analysis of variance (ANOVA) or two-tailed Student’s *t*-test with GraphPad Prism 4 software. *P* < 0.05 was statistically significant.

## Results

### Lipid metabolism

**Figure 1:**
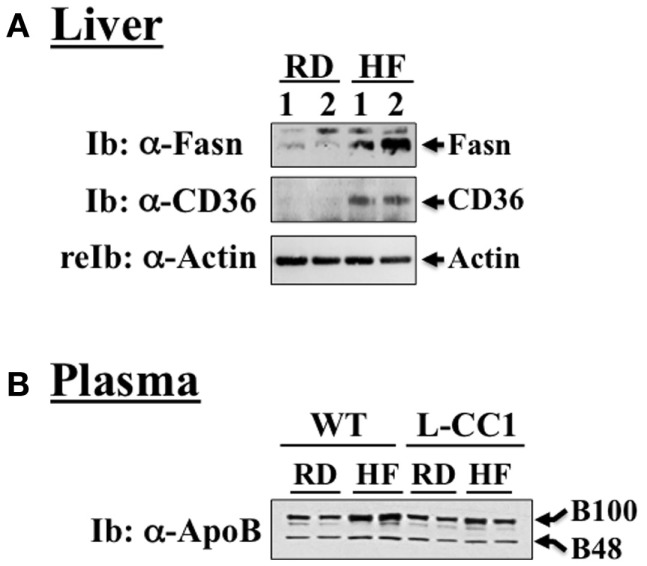
**Western analysis of proteins involved in hepatic lipid homeostasis**. **(A)** Liver lysates from wild-type mice fed a regular (RD) or a high-fat diet (HF) for 4 months were analyzed by immunoblotting with α-Fasn and α-CD36 antibodies, followed by reprobing with α-actin for normalization. A representative gel of three different experiments performed on two mice per feeding group is included. **(B)** Plasma from both WT and L-CC1 mice was diluted and analyzed by 4–10% gradient SDS-PAGE and immunoblotting with an antibody against apolipoprotein B (ApoB), which recognizes both ApoB48 and ApoB100. A representative gel of two independent experiments performed on two different pairs of mice per feeding group per genotype is included.

As expected, HF diet caused hyperinsulinemia and fed hyperglycemia (Table 2), markers of insulin resistance, in WT mice. In support of the positive effect of hyperinsulinemia on lipogenesis (4), qRT-PCR (Table 3) and Western blot analysis showed increased mRNA and protein levels of Fasn, respectively, in the liver of HF-fed WT mice relative to mice fed a regular diet (RD) (Figure 1A). HF diet also induced hepatic mRNA (Table 3) and protein levels of CD36 fatty acid translocase (Figure 1A), in addition to inducing the mRNA level of fatty acid transport protein-1 (Fatp-1) (Table 3). Together with increased lipogenesis, elevated lipid transport could contribute to increased hepatic triacylglycerol content in HF-fed WT mice (Table 2).

**Table 2:** **Effect of high-fat diet for 4 months on plasma and tissue biochemistry**.

	WT		L-CC1	
	RD	HF	RD	HF
Body weight (BW) (g)	26.0 ± 0.80	34.0 ± 1.12*	24.0 ± 1.03	34.0 ± 2.32*
Body length (cm)	9.73 ± 1.52	10.5 ± 2.21	9.70 ± 1.00	10.4 ± 1.77
Visceral adipose tissue (% BW)	2.45 ± 0.25	7.55 ± 0.26*	1.36 ± 0.26^†^	5.76 ± 0.48*^†^
Brown adipose tissue (% BW)	0.39 ± 0.05	0.39 ± 0.03	0.34 ± 0.03	0.36 ± 0.04
Subcutaneous fat (% BW)	1.76 ± 0.12	6.23 ± 0.51*	1.18 ± 0.13^†^	5.02 ± 0.65*
Fasting plasma insulin (pM)	60.0 ± 1.43	162.0 ± 8.15*	58.2 ± 2.14	78.3 ± 3.34*^†^
Fed blood glucose (mg/dl)	120.0 ± 1.42	150.0 ± 3.52*	124.0 ± 3.25	130.0 ± 2.82^†^
Hepatic triacylglycerol (μg/mg protein)	122.0 ± 5.00	505.0 ± 20.4*	135.0 ± 3.40	152.0 ± 14.0^†^
Fasting plasma triacylglycerol (mg/dl)	65.4 ± 3.82	75.3 ± 4.60	50.3 ± 3.22^†^	58.4 ± 1.42^†^
Fasting plasma NEFA (mEq/l)	0.62 ± 0.04	0.92 ± 0.03*	0.58 ± 0.02	0.64 ± 0.04^†^

*Mice (*n* > 8/feeding group/genotype) were fed an HF diet for 4 months starting at 2 months of age. Fat depots were excised and weighed. Intra-abdominal visceral adipose tissue fat refers to combined weight of mesenteric and gonadal fat. The relative mass of fat depots was expressed as percentage of body weight (% BW)*.

*Values are expressed as mean ± SEM*.

*P < 0.05 HF vs. RD per genotype.

*^†^*P* < 0.05 L-CC1 vs. WT per feeding group*.

**Table 3:** **Effect of high-fat diet on mRNA levels of lipid metabolism genes in liver**.

	WT		L-CC1	
	RD	HF	RD	HF
Fasn	1.25 ± 0.12	3.60 ± 0.40*	1.00 ± 0.20	1.45 ± 0.25^†^
Cd36	1.23 ± 0.11	2.00 ± 0.30*	0.67 ± 0.12	0.86 ± 0.12^†^
Fatp-1	1.19 ± 0.15	2.33 ± 0.10*	1.18 ± 0.05	0.87 ± 0.08^†^

*Tissue mRNA levels were analyzed in duplicate per mouse (*n* ≥ 6 mice/feeding group/genotype)*.

*Values were normalized to 18S and expressed as mean ± SEM*.

*P < 0.05 HF vs. RD per genotype.

*^†^*P* < 0.05 L-CC1 vs. WT per feeding group*.

Increased production of hepatic triacylglycerol drives output, as supported by elevated plasma apolipoprotein B (ApoB48/ApoB100) protein levels in WT mice (Figure 1B). With fasting plasma triacylglycerol levels being intact (Table 2), it is likely that triacylglycerol was redistributed to the WAT, as expected from mice on the C57/BL6J genetic background that favors substrates partitioning to the adipose tissue (6). Supporting this notion, mRNA levels of Fatp-4 and lipoprotein lipase (Lpl) that are critical in lipid transport to the adipocyte were elevated in HF-fed relative to RD-fed WT mice (Table 4). This appears to drive adipogenesis, as suggested by higher Srebp-1c mRNA levels (Table 4), and ultimately, increased visceral obesity (Table 2). Elevated mRNA levels of hormone sensitive lipase (Hsl) (Table 4) and the rise in fasting plasma NEFA levels (Table 2) are consistent with increased lipolysis in HF-fed WT mice.

**Table 4:** **Effect of 4 months of high-fat diet on mRNA of genes in white adipose tissue**.

	WT		L-CC1	
	RD	HF	RD	HF
Fatp-4	0.80 ± 0.10	1.80 ± 0.22*	0.96 ± 0.24	0.55 ± 0.11^†^
Lpl	1.20 ± 0.21	3.51 ± 0.30*	1.12 ± 0.12	1.25 ± 0.40^†^
Srebp-1c	1.09 ± 0.13	4.03 ± 0.70*	1.26 ± 0.51	0.94 ± 0.32^†^
Hsl	0.49 ± 0.10	0.80 ± 0.11*	0.51 ± 0.13	0.27 ± 0.14^†^
F4/80	8.85 ± 4.50	21.8 ± 2.61*	5.45 ± 1.22	9.08 ± 3.01*^†^
TNFα	2.75 ± 1.37	6.56 ± 0.78*	3.55 ± 0.78	3.01 ± 1.05^†^
Smad7	11.3 ± 2.56	1.72 ± 0.40*	15.2 ± 2.05	9.40 ± 2.04^†^
Col6α3	0.67 ± 0.11	1.58 ± 0.25*	0.58 ± 0.17	0.48 ± 0.13^†^
α-Sma	0.03 ± 0.01	1.05 ± 0.33*	0.06 ± 0.01	0.19 ± 0.05^†^

*Mice (*n* > 5 per feeding group per genotype) were fed an HF diet for 4 months starting at 2 months of age*.

*Tissue mRNA levels were analyzed in duplicate per each mouse and normalized to 18S*.

*Values are expressed as mean ± SEM*.

*P < 0.05 HF vs. RD per genotype.

*^†^*P* < 0.05 L-CC1 vs. WT per feeding group*.

In L-CC1 mice, however, hepatic triacylglycerol content was neither modified by HF intake (Table 2) nor were mRNA levels of Fasn, Fatp-1, and Cd36 (Table 3). Consistent with normal hepatic lipogenesis, plasma ApoB48/ApoB100 protein levels in L-CC1 mice were not significantly altered by HF diet (Figure 1B). Moreover, HF did not modulate the mRNA level of genes involved in lipid metabolism in the adipocyte (Lpl, Fatp-4, and Srebp-1c) (Table 4). Consistent with normal fasting plasma NEFA (Table 2), HF did not alter Hsl mRNA levels in the WAT of L-CC1, as it did to WT mice (Table 4).

### Visceral obesity

**Figure 2:**
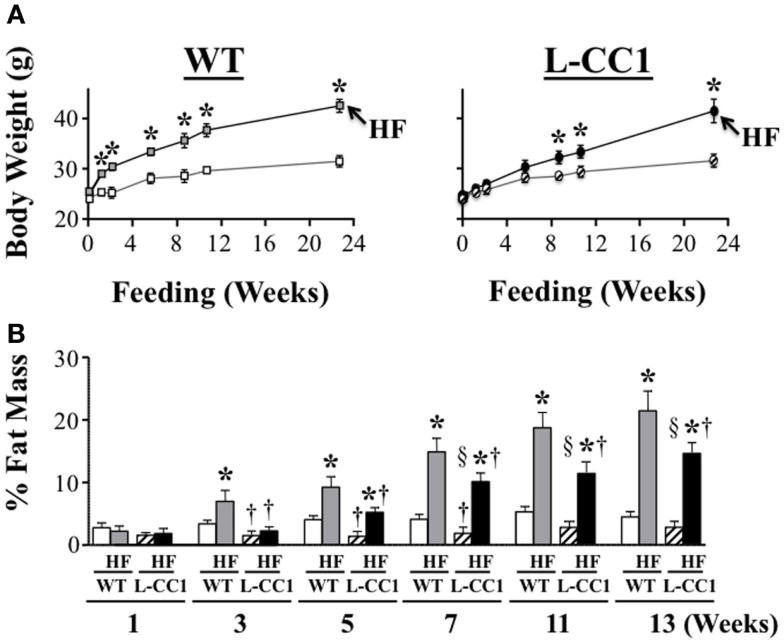
**Time-dependent effect of high-fat diet on fat distribution**. **(A)** Mice (*n* > 10 mice/feeding group/genotype) were fed a regular (RD) or a high-fat (HF) diet and their body weight (grams) was weighed for 0–24 weeks. Values expressed as mean ± SEM. *P < 0.05 HF vs. RD and ^†^*P* < 0.05 L-CC1 vs. WT per feeding group. **(B)** Whole body fat mass was determined using NMR and expressed as percentage of total body weight (*n* > 10 mice/feeding group/genotype). Values expressed as mean ± SEM. *P < 0.05 HF vs. RD, ^†^*P* < 0.05 L-CC1 vs. WT per feeding group and ^§^*P* < 0.05 HF-fed L-CC1 vs. RD-fed WT mice.

High-fat feeding time dependently increased body weight gain in WT mice (Figure 2A). In contrast, it took ≥6 weeks before HF induced a statistically significant body weight gain in L-CC1 (Figure 2A). NMR analysis (Figure 2B) revealed persistently lower fat mass in L-CC1 relative to WT mice under normal feeding conditions until ~5 months of age, at which point this difference became statistically insignificant (Figure 2B, starting at about 11 weeks of experimental feeding). In contrast to total fat mass, visceral (mostly gonadal) and subcutaneous fat mass remained lower in RD-fed L-CC1 than RD-fed WT mice even until 6 months of age (Table 2).

While HF feeding caused an increase in total fat mass relative to RD feeding in both mouse groups, this effect was delayed (at 5 vs. 3 weeks in L-CC1 vs. WT) (Figure 2B). Furthermore, fat accumulation in L-CC1 mice did not become significantly higher than RD-fed WT until after 7 weeks of HF feeding but remained lower than HF-fed WT even after 4 months of HF, as shown by NMR (Figure 2B), and by ^1^H-magnetic resonance spectroscopy (7). After 4 months of HF diet, visceral fat mass remained lower in L-CC1 than WT mice, while subcutaneous fat mass became comparable in both groups of mice (Table 2).

### Increased macrophage recruitment to white adipose tissue in response to high-fat diet

**Figure 3:**
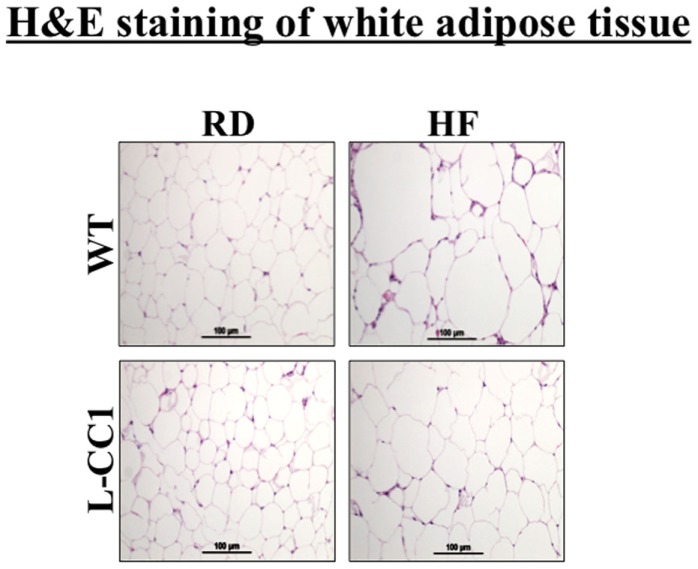
**Histological analysis of white adipose tissue**. White adipose tissue histology was assessed in H&E stained sections (*n* > 4 mice/feeding group/genotype). Lipid droplets indicate adipocytes’ expansion in response to high-fat (HF) relative to regular diet (RD) in both genotypes. Of note, the expansion in L-CC1 was relatively weaker than in WT mice. Representative images from three sections per mouse are shown.

Histological analysis of H&E stained sections from WAT showed a significant expansion of adipocytes in both groups of mice in response to 4 months of HF intake, but to a lower extent in L-CC1 mice (Figure 3).

**Figure 4:**
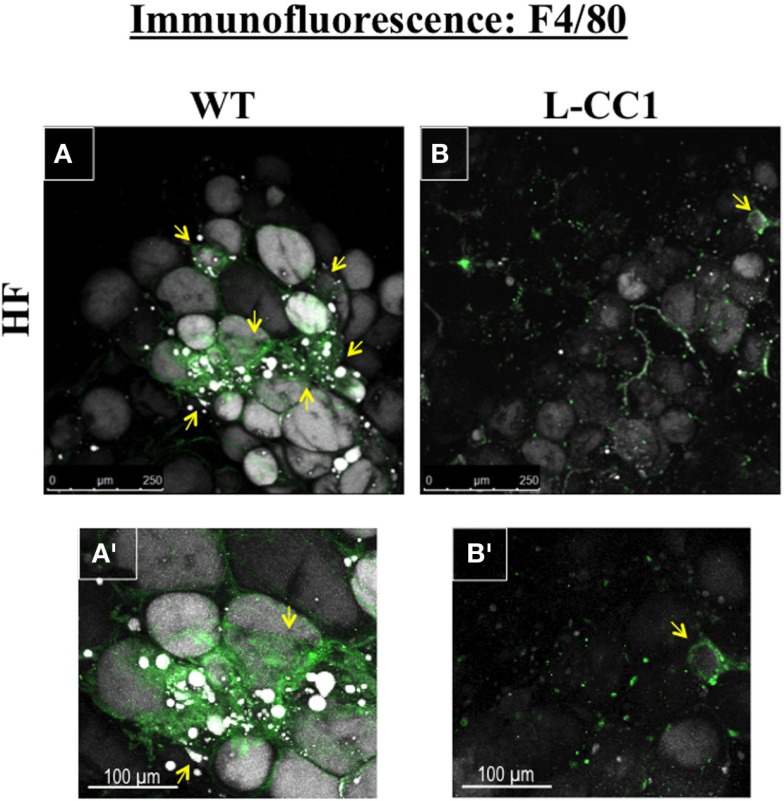
**Immunofluorescence analysis of white adipose tissue**. Whole white adipose tissue from HF-fed WT and L-CC1 mice was stained with BODIPY 558/568 to detect lipid (gray) and with anti-F4/80 to detect macrophages (green). All images were captured using LSCM with a 20× objective and are 2D projections of a 3D image *z*-stack. **(A)** Representative picture from HF-fed WT mice. Yellow arrows show multiple areas with crown-like structures (CLS). **(A’)**. A blow up of the area around the bottom left yellow arrow in (A) showing multiple layers of CLS containing macrophages (green) surrounding adipocytes (gray). Also visible are small pieces of lipid inside the macrophages. **(B)** Representative projection from HF-fed L-CC1 mice. One yellow arrow points out the single CLS detected in the image. **(B’)**. A blow up of the single CLS in (B) (yellow arrow) surrounding one adipocyte from HF-fed L-CC1 mice. No small pieces of lipid are visible inside of macrophages. Representative images from three sections per mouse are shown.

Moreover, immunofluorescence analysis of F4/80, a macrophage marker, showed multiple crown-like structures (CLS) containing macrophages in WAT from HF-fed WT mice (Figures 4A,A’, green). As previously shown (14), moving through the individual *z*-stack sections shows numerous small pieces of lipid inside the macrophages in CLS (Figures 4A,A’), indicating adipocyte degradation in HF-fed WT mice. In contrast, HF-fed L-CC1 mice exhibited minimal CLS formation with little evidence of lipids inside the macrophages in these structures. Consistently, HF diet-induced F4/80 mRNA levels by ~2.5-fold in WT as compared to ~1.5-fold in WAT of L-CC1 mice, and mRNA levels of TNFα adipokine in WT mice (by ~2-fold), but not in L-CC1 mice (Table 4). Together, this demonstrates that protecting hepatic CEACAM1 levels against HF diet limited the development of inflammation in the WAT of L-CC1 mice.

### Induction of fibrosis and TGF-β activation in white adipose tissue by high-fat diet

**Figure 5:**
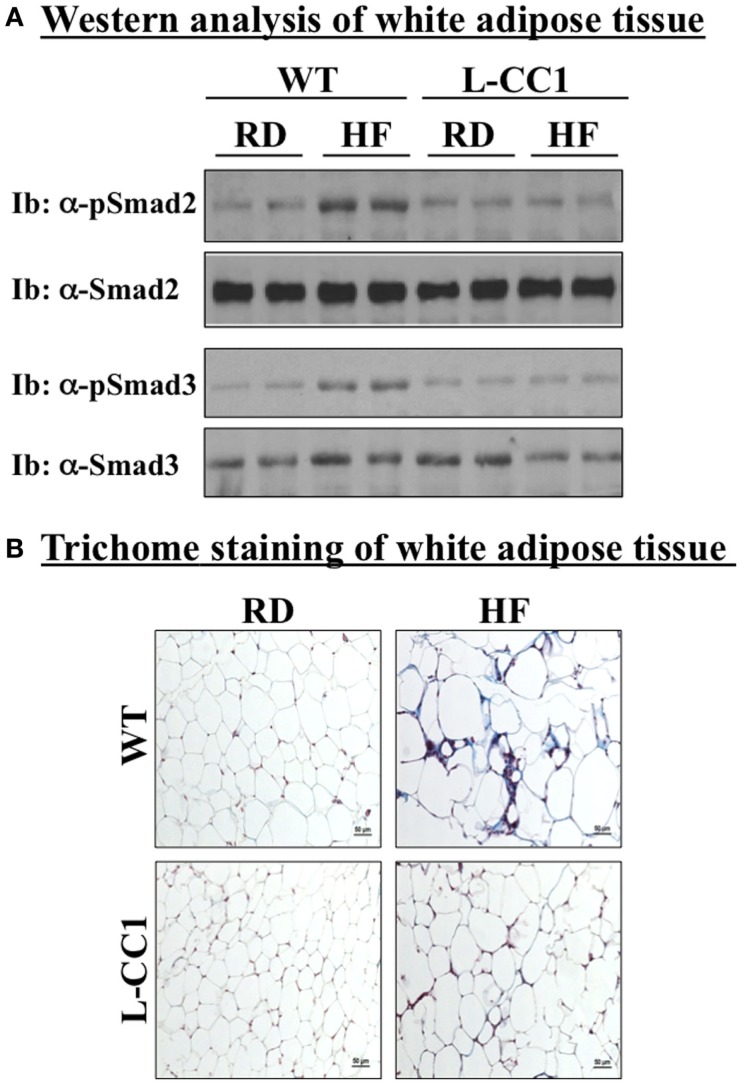
**Fibrosis in white adipose tissue**. **(A)** Lysates from white adipose tissue of wild-type (WT) and L-CC1 mice fed a regular (RD) or a high-fat diet (HF) for 4 months were analyzed by immunoblotting with α-phospho-Smad 2 and phospho-Smad 3 antibodies followed by reimmunoprobing (reIb) with antibodies against total Smad 2 and 3, respectively, for normalization. A representative gel of three experiments performed on two different mice per feeding group is included. **(B)** White adipose tissue from five mice/feeding group/genotype was analyzed by trichrome staining to detect collagen deposition. Representative images from three sections per mouse are shown.

In agreement with reports showing TNFα inhibition of the expression of Smad 7 (15), a negative regulator of TGF-β–Smad2/3 signaling pathway (16, 17), HF markedly repressed (by ~5-fold) the mRNA level of Smad 7 in WT, but not L-CC1 mice (Table 4). Subsequently, HF induced Smad 2 and Smad 3 activation in the WAT of WT, but not L-CC1 mice, as assessed by Western analysis of Smad 2 and Smad 3 phosphorylation (Figure 5A).

In adipose tissue derived from WT, but not L-CC1 mice, HF induced significantly the transcript levels of two main players in fibrosis (Table 4): α-smooth muscle actin (α-Sma) (by ≥10-fold) and endotrophin/Col6α3 (by ~2-fold), a protein that promotes metabolic derangement and fibrosis in adipose tissue (18). This translated into a higher induction of collagen deposition in the WAT of WT than L-CC1 mice by HF feeding, as shown by trichrome staining (Figure 5B).

## Discussion

Phenocopying L-SACC1 mice with liver-specific inactivation of CEACAM1 and global *Cc1^−/−^* null mice (1–3), HF diet represses hepatic CEACAM1 levels to impair insulin clearance and cause hyperinsulinemia, which in turn, activates *de novo* lipogenic pathways and elevates lipid production in liver (7). The current studies show that this drives lipid output (manifested by a rise in plasma ApoB100/ApoB48) and redistribution to WAT to provoke visceral obesity. This is consistent with a positive correlation between liver steatosis, hyperinsulinemia, and high plasma ApoB levels in humans and rodents (19–23). For instance, mice with conditional null mutation of the insulin receptor in liver exhibit impairment of insulin clearance and hepatic insulin resistance with elevated lipogenesis, and a rise in plasma ApoB100/ApoB48 levels in parallel to low plasma triacylglycerol levels and obesity (24).

In support of the important role that reducing hepatic CEACAM1 level plays in the pathogenesis of diet-induced metabolic derangement, protecting hepatic CEACAM1 levels by means of transgenic induction preserves insulin clearance and prevents insulin resistance and hepatic steatosis in response to HF feeding (7). The current studies show that it also causes a delay in the progression of fat accumulation and limits the expansion of adipocytes in response to HF diet. Together, this supports an important role for altered CEACAM1-dependent insulin clearance pathways in the pathogenesis of diet-induced hepatic steatosis and visceral obesity. These findings lend a mechanistic underpinning for the marked reduction of hepatic CEACAM1 observed in insulin-resistant obese subjects (25).

Immunofluorescence analysis showed less recruitment of CLS-containing macrophages to the WAT of L-CC1 and less adipocyte degradation inside these macrophages compared to WT mice. In addition to normal mRNA levels of TNFα, an adipokine that blunts insulin action (26), this could explain the protected insulin sensitivity along the liver-WAT axis (7).

In addition to inflammation (26), visceral obesity and insulin resistance are associated with increased fibrosis and activation of TGF-β signaling pathways in WAT in rodents (18, 27) and humans (28). Consistently, HF feeding induced more collagen deposition and activation of the TGF-β–Smad 2/3 pathway (16, 17) in WT than L-CC1 mice. This could prevent adipose tissue remodeling and contribute to the more metabolic derangement in HF-fed WT mice (29).

In summary, the current studies demonstrate that gain-of-function of CEACAM1 in liver restricts visceral obesity caused by HF diet, and that this is mediated, at least in part, by reducing lipid output from liver and limiting inflammation and fibrosis in WAT. Together with increased FGF21 production (7), this could mediate in part, preserved energy expenditure in L-CC1 mice (7, 28, 30). Because CEACAM1 protein is not produced to a significant extent in WAT (12), preserving adipose tissue biology could be attributed to extra-adipocytic factors caused by inducing CEACAM1 expression in liver. Although the underlying mechanisms are not fully delineated, the current studies provide a proof-of-principle of the importance of hepatic CEACAM1-dependent insulin clearance in the regulation of visceral obesity, and adipose tissue inflammation and fibrosis in response to HF diet.

## Author Contributions

SL researched data, designed experiments, and wrote a first draft of the manuscript, LR, SG, SK, EE, TB, GH, and QA researched data. AD designed and generated the targeting vector for the generation of the L-CC1 mouse line, screened and propagated the mouse line, and reviewed the manuscript. WP generated the L-CC1 mouse. MM participated in scientific discussions, in detecting CLS, and in the revision of the manuscript. SN was responsible for study design, conceptualization, data analysis and results interpretation, reviewing, and revising the manuscript. SN had full access to all the data of the study and takes responsibility for the integrity and accuracy of data analysis and the decision to submit and publish the manuscript.

## Conflict of Interest Statement

The authors declare that the research was conducted in the absence of any commercial or financial relationships that could be construed as a potential conflict of interest.

## Abbreviations

α-Sma, alpha-smooth muscle actin; ApoB48/ApoB100, apolipoprotein B; Col6α3; alpha 3 chain of collagen 6-also known as endotrophin; *Cc1^–/–^*, global *Ceacam1* null mouse; CEACAM1, carcinoembryonic antigen-related cell adhesion molecule 1; *Ceacam1*, gene encoding CEACAM1 protein; Fasn, fatty acid synthase; FAT/CD36, fatty acid translocase/cluster of differentiation protein 36; FATP, fatty acid transporter protein; HF, high-calorie high-fat diet; Hsl, hormone-sensitive lipase; L-CC1, mice with liver-specific over-expression of wild-type CEACAM1; Lpl, lipoprotein lipase; L-SACC1, liver-specific S503A CEACAM1 mutant mouse; NEFA, non-esterified fatty acids; RD, regular diet; SREBP-1c, sterol regulatory element-binding protein; TNFα, tumor necrosis factor alpha; TGF-β transforming growth factor beta.
